# Ultracompact meta-pixels for high colour depth generation using a bi-layered hybrid metasurface

**DOI:** 10.1038/s41598-019-51946-8

**Published:** 2019-10-25

**Authors:** Jeong-Geun Yun, Jangwoon Sung, Sun-Je Kim, Hansik Yun, Chulsoo Choi, Byoungho Lee

**Affiliations:** Inter-University Semiconductor Research Center and School of Electrical and Computer Engineering, Seoul National University, Gwanak-Gu Gwanakro 1, Seoul, 08826 Korea

**Keywords:** Displays, Metamaterials

## Abstract

Nano-structural colour pixels have attracted much attention as promising solutions for compact display devices. However, it is difficult to miniaturize and integrate conventional transmissive colour filtering components for high resolution pixels within subwavelength scale without sacrificing colour depth. Here, we propose a novel colour pixel structure using bi-layered hybrid metasurfaces that are composed of aluminum nanograting and amorphous silicon nanorod layers. The independent high-contrast control of resonance intensity and spectral position is achieved by anisotropic Mie resonances and cavity effect between stacked two metasurfaces. Moreover, the proposed structures permit the wide colour gamut through the novel physical principles. In addition, a meta-pixel which can provide gradual tuning of colour is demonstrated to obtain high colour depth. The proposed structures are expected to be fruitful for the development of next generation display and imaging systems.

## Introduction

Nowadays, high-end display systems are faced with the demands for compact form factors, small pixel sizes, and wide colour gamut. However, such demands have not been completely satisfied due to the difficulties of miniaturization and integration of the conventional optical elements such as colour filters and polarizers. Recently, plasmonic colour generations have been widely studied to generate colours at nanoscales. A wide range of colour generation has been demonstrated by tuning the plasmonic resonance of the metallic antenna array with various metals such as silver^1–4^ and aluminum^5–8^. However, they provide broad spectral response and low saturation colours due to intrinsic loss of the metals. Thus, to achieve highly saturated colours with high purity, dielectric metasurfaces based on the resonances in high index dielectric nanoantennas have been proposed^9–13^. High index dielectric materials such as silicon can support electric and magnetic resonances, also called Mie resonances^14,15^ and their scattering characteristics are determined by the interferences between the resonances. By manipulating the Mie resonances, pure colour generation has been presented in reflection type dielectric metasurfaces^9–12^. However, transmission type dielectric metasurfaces suffer from low saturation colours because it is difficult to provide narrow resonance bandwidth over the entire visible wavelengths by designing the multipolar electric and magnetic resonances of dielectric structures^11–13^. In addition, the aforementioned structural colour generations have common limitations of low colour depth (~thousands of colours). This is because the spectral tuning relies on tiny adjustment of the length parameters such as a radius, period and distance between the nanostructures. This approach makes it difficult to obtain the colour pixels that are expressed in a desired colour with a specific hue, saturation, and value because the resonance wavelength and intensity are changed simultaneously by adjusting the length parameters^16,17^. As a result, the limited number of colours drastically degrades a resolution of a real picture and leads to limited applications in imaging and display systems.

Here, we propose a novel structure of bi-layered hybrid metasurfaces to achieve highly saturated colours and high colour depth generation with ultracompact form factors. The proposed bi-layered metasurfaces consisting of aluminum (Al) nanograting and anisotropic amorphous silicon (a-Si) nanorod layers can provide independent control of spectral resonance and intensity by adjusting different geometric parameters. The hybrid metasurfaces only allow transmission of specifically designated resonance peaks mainly determined by size of the nanorod. In addition, an anisotropic cavity effect between the nanograting and nanorod layers permits highly saturated colour generation. This is because the spectral broadening caused by the electric and magnetic multipolar resonances in the dielectric nanorods can be ameliorated by manipulating the interferences among those resonances through the adjustment of the distance between the two layers. Moreover, based on the proposed structures, the method for gradual colour tuning and high colour depth generation is presented using additive subwavelength colour mixing. Spatially varying unit cell structures of the proposed hybrid metasurfaces, called meta-pixels, allow to design the spectral resonance and intensity. Through this colour mixing method, myriad of colour pixels having desired hue, saturation, and value can be created within subwavelength thickness, and thus it could be applied to reproduce real picture images with high colour quality. Therefore, the bi-layered hybrid metasurfaces can be a powerful solution for extreme miniaturization of many imaging and display systems.

## Principles of Bi-layered Hybrid Metasurfaces

**Figure 1:**
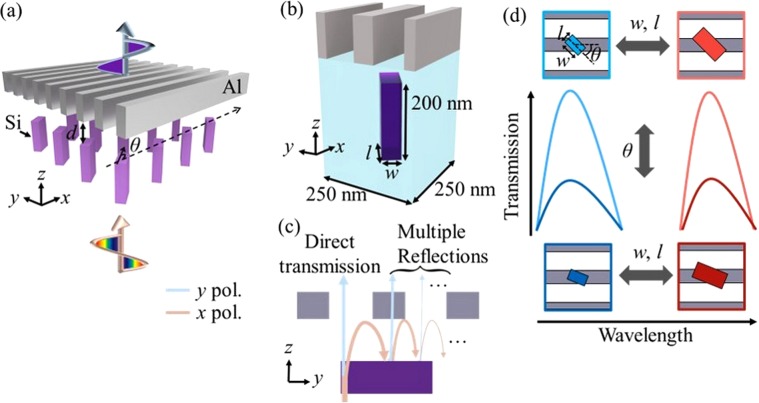
(**a**) Artistic rendering of the bi-layered hybrid metasurface. (**b**) Unit cell structure of the proposed structure. The period, thickness, and width of the nanograting are 125 nm, 40 nm, and 120 nm, respectively. (**c**) The unit cell structure on the *y*-*z* plane. (**d**) Independent control of the spectral intensity and wavelength. The intensity and wavelength of the transmitted light can be controlled by the rotated angle (*θ*) and size (*w*, *l*) of the nanorods, respectively.

Figure 1a,b show schematic illustrations of the bi-layered hybrid metasurfaces composed of Al nanograting layer and rotated a-Si nanorod layer. Here, the nanograting layer is designed to act like a linear polarizer over the entire visible range, and the nanorods rotated by *θ* are separated by *d* from the nanograting layer. When the *x*-polarized white light is incident on the bi-layered metasurface, the rotated nanorods rotate, depending on *θ*, the polarization angle of the incidence light at the resonances of the nanorods. The *y*-polarized component of the rotated light can pass through the nanograting layer, but the *x*-polarized component does not penetrate. Thus, the *x*-polarized component of the incident *x*-polarized light undergoes multiple reflections between the nanograting and nanorods until it is reflected back to −*z* direction or converted to *y*-polarized light by the nanorods as shown in Fig. 1c. That is, except for the *y*-polarized lights converted by the polarization rotations of the nanorods, the incident white light is reflected back or absorbed by the structures.

The spectral characteristics of the rotated nanorod layer can be related to the polarizability tensor of the electric ***a*** and magnetic ***b*** multipolar resonances which are determined by dispersion of the materials and their geometric parameters^18^. When the nanorods are anisotropic and small enough compared to the wavelength of interest, electric dipole (ED) and magnetic dipole (MD) are dominantly induced and the polarizability tensors can be reduced to $${\bf{a}}=(\begin{array}{cc}{a}_{xx} & 0\\ 0 & {a}_{yy}\end{array})$$, and $${\bf{b}}=(\begin{array}{cc}{b}_{xx} & 0\\ 0 & {b}_{yy}\end{array})$$. For the nanorods smaller than the wavelengths of the incident light, the nanorod layer can be modeled as the effective surface with averaged transverse polarization currents^19^, and their transmission and reflection matrices can be calculated as the following equations:

1 $$t=(\begin{array}{cc}1-\frac{i\omega }{2{p}_{x}{p}_{y}}(\frac{{\eta }_{0}{a}_{xx}}{1-{a}_{xx}{C}_{xx}^{e}}+\frac{1}{{\eta }_{0}}\frac{{b}_{xx}}{1-{b}_{xx}{C}_{xx}^{m}}) & 0\\ 0 & 1-\frac{i\omega }{2{p}_{x}{p}_{y}}(\frac{{\eta }_{0}{a}_{yy}}{1-{a}_{yy}{C}_{yy}^{e}}+\frac{1}{{\eta }_{0}}\frac{{b}_{yy}}{1-{b}_{yy}{C}_{yy}^{m}})\end{array}),$$

2 $$r=(\begin{array}{cc}\frac{i\omega }{2{p}_{x}{p}_{y}}(-\frac{{\eta }_{0}{a}_{xx}}{1-{a}_{xx}{C}_{xx}^{e}}+\frac{1}{{\eta }_{0}}\frac{{b}_{xx}}{1-{b}_{xx}{C}_{xx}^{m}}) & 0\\ 0 & \frac{i\omega }{2{p}_{x}{p}_{y}}(-\frac{{\eta }_{0}{a}_{yy}}{1-{a}_{yy}{C}_{yy}^{e}}+\frac{1}{{\eta }_{0}}\frac{{b}_{yy}}{1-{b}_{yy}{C}_{yy}^{m}})\end{array}),$$

where *p*_*x*_ and *p*_*y*_ are periods, *ω* is angular frequency, *η*_0_ is the free space impedance, *C* is an interaction coefficient, and the superscript *e* and *m* mean electric and magnetic, respectively. As shown in Eqs 1 and 2, the transmission and reflection coefficients are combinations of ED and MD derived components. For convenience, the components derived from ED and MD are defined as ${\alpha }_{i}=\frac{i\omega }{2{p}_{x}{p}_{y}}\frac{{\eta }_{0}{a}_{ii}}{1-{a}_{ii}{C}_{ii}^{e}}$ and ${\beta }_{i}=\frac{i\omega }{2{p}_{x}{p}_{y}}\frac{1}{{\eta }_{0}}\frac{{b}_{ii}}{1-{b}_{ii}{C}_{ii}^{m}}$, respectively. For simplicity, we here assume that the nanograting acts like a perfect linear polarizer and higher order multiple reflection terms are neglected (See Section 1 of Supplementary Information for the details of the derivation). Then, total transmitted complex electric field *E*_*tot*_ of the bi-layered hybrid metasurface for *x*-polarized incident light can be decomposed by the ED and MD components as following equations:

3 $${{\boldsymbol{E}}}^{e}={e}^{ik{n}_{1}d}\,\sin \,\theta \,\cos \,\theta ({\alpha }_{y}-{\alpha }_{x})[1+{e}^{i2k{n}_{1}d}(1-{\cos }^{2}\theta ({\alpha }_{x}+{\beta }_{x})-{\sin }^{2}\theta ({\alpha }_{y}+{\beta }_{y}))]\bar{y},$$

4 $${{\boldsymbol{E}}}^{m}={e}^{ik{n}_{1}d}\,\sin \,\theta \,\cos \,\theta ({\beta }_{y}-{\beta }_{x})[1-{e}^{i2k{n}_{1}d}(1-{\cos }^{2}\theta ({\alpha }_{x}+{\beta }_{x})-{\sin }^{2}\theta ({\alpha }_{y}+{\beta }_{y}))]\bar{y},$$

5 $${{\boldsymbol{E}}}_{tot}={{\boldsymbol{E}}}^{e}+{{\boldsymbol{E}}}^{m},$$

where *k* is the free space wavenumber, *n*_1_ is the refractive index of the spacer medium, and $\bar{y}$ is unit vector along with *y*-axis, *E*^*e*^ and *E*^*m*^ are complex electric fields caused by ED and MD resonances, respectively. The first and second terms in the square bracket of Eqs 3 and 4 indicate the direct and indirect transmission components, respectively (cf. Fig. 1c). Also, it is apparent that anisotropic response of ED *(α*_*y*_ − *α*_*x*_) and MD *(β*_*y*_ − *β*_*x*_) resonances permit the incident light to penetrate the proposed structure as converting the incident light to *y*-polarized light, and the amplitude can be controlled by the rotated angle of the nanorod *θ*. It is also noticeable that the transmitted light is related not only to the amplitude and phase of both ED and MD resonances but also to the cavity terms governed by *d*. This indicates that the proposed structure gives an additional degree of freedom for tuning the spectral characteristics by adjusting *d*, and thus it could be fruitful to provide wide gamut of the response colours. Based on this scenario, it is possible that the spectral resonance and intensity are independently controlled by the different geometric parameters, size (*w*, *l*) and *θ*, respectively, as shown in Fig. 1d.

**Figure 2:**
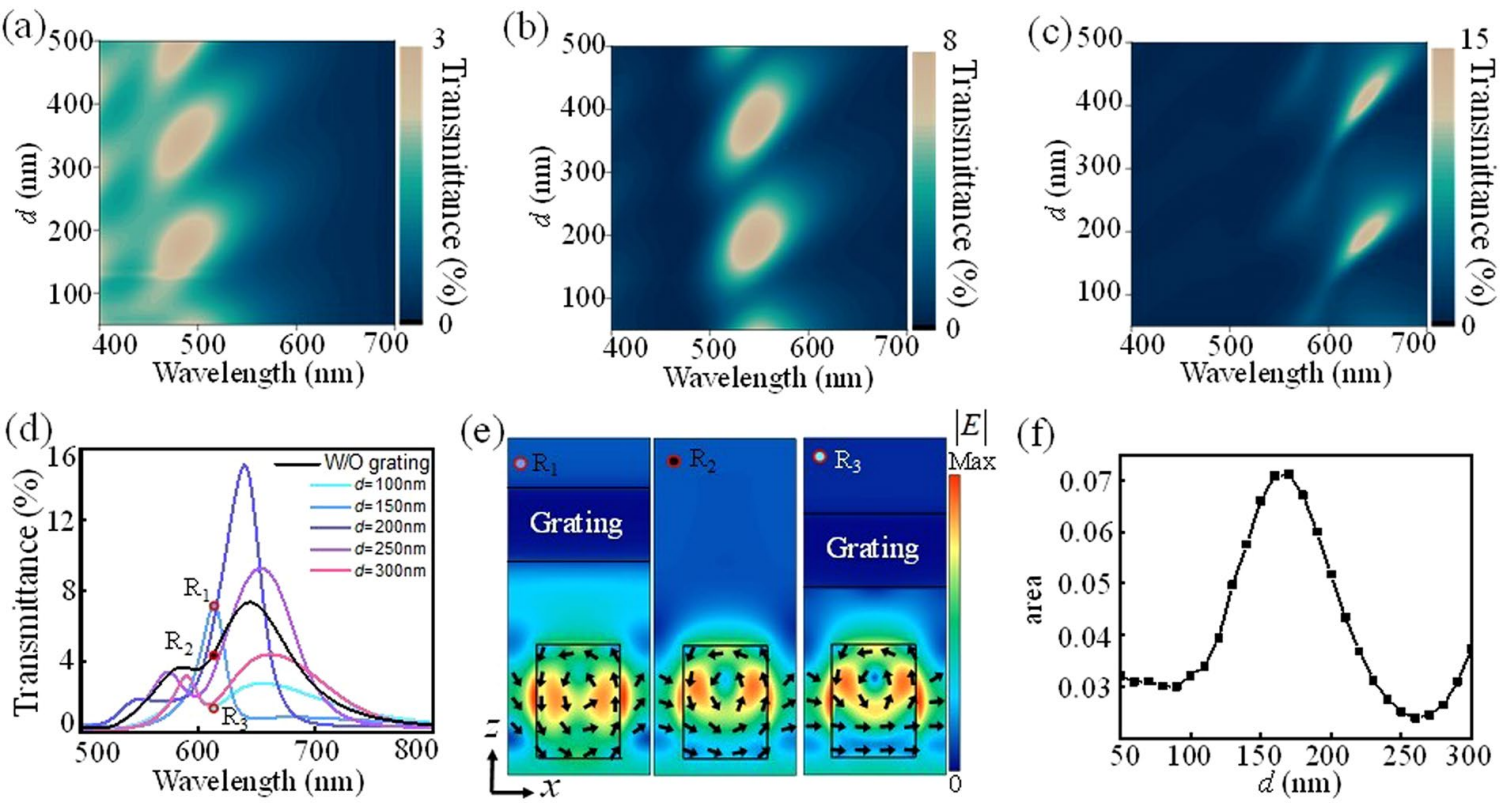
Transmittance spectra of the bi-layered hybrid metasurfaces having various nanorods of (**a**) *w* = 30 nm, *l* = 80 nm, (**b**) *w* = 60 nm, *l* = 90 nm, (**c**) *w* = 100 nm, *l* = 120 nm and fixed *θ* = 45° as changing *d*. (**d**) The transmittance spectra of the various structures with *w* = 100 nm, *l* = 120 nm, and *θ* = 45°. For *x*-polarized incident light, the transmitted *y*-polarized light of the nanorod layer without the nanograting (black line) and the transmittance of the bi-layered structures with varying *d* (colour lines) are calculated. (**e**) The electric field distributions of various structures represented as R_1_, R_2_, and R_3_. The R_1_ and R_3_ have the nanograting layer with *d* = 150 nm and 100 nm, respectively, and R_2_ does not have the nanograting layer. (**f**) The area of the RGB colour space of the bi-layered structures with various *d*.

To understand the cavity effect in the bi-layered hybrid metasurface structure, the transmittance spectra of the structures having various *w* and *l* are calculated as changing *d* as shown in Fig. 2a–c. For the numerical calculations, a commercial finite-difference time-domain tool (Lumerical Solutions, Inc.) is used. The proposed bi-layered metasurface is modeled as an Al nanograting layer stacked on top of the SiO_2_ substrate, and the a-Si nanorod is embedded in the substrate (see Fig. 1b). The spectral responses are calculated under the normal incidence of broadband plane wave on the substrate side, and the propagation direction is set to +*z*. Dielectric constants of Al and SiO_2_ are taken from a textbook^20^, and dielectric constant of the fabricated a-Si thin film is experimentally measured and exploited to our simulations. Since Mie resonances are excited at the longer wavelengths as the sizes of nanorods increase, the spectral resonances exhibit a red shift when the size increases. As predicted in Eqs 3–5, it can be seen that the transmittances of the structures are periodically suppressed or enhanced depending on *d* due to the cavity effect. Moreover, it is noticeable that the resonance wavelengths and bandwidths of the structures are fluctuated as changing *d*. To investigate this tendency, the spectra and electric field distributions of the bi-layered structures for various *d* values are compared with those of the rotated nanorod layer without the nanograting. Figure 2d shows the transmittance of the *y*-polarized light of the nanorod layer without the nanograting (black solid line). As can be seen, the nanorod layer without the nanograting layer exhibits broad spectral response due to the electric and magnetic resonances. In contrast, the spectral bandwidth and resonance of the proposed bi-layered structures are fluctuated by *d* because the interferences between the Mie resonances can be adjusted through the reflection components (cf. Eqs 3 and 4). In particular, the cavity effect of the bi-layered structures leads to the significant narrower full width at half maximum (FWHM) of 26 nm compared to the FWHM of the nanorod layer without the nanograting (106 nm). To understand the cavity effect more concretely, the electric field distributions of various structures (*d* = 150 nm (R_1_), without the nanograting (R_2_), and *d* = 100 nm (R_3_)) at the same wavelength of 612 nm is calculated as shown in Fig. 2e. For R_1_, the MD resonance is dominantly excited in the nanorod, and the transmittance peak occurs at 612 nm. As the structure changes from R_1_ to R_3_, the ED resonance becomes stronger, and thus the transmittance decreases due to the destructive interference between the ED and MD components. This destructive interference leads to transmittance dip for R_3_ through enhancing the ED resonances and strongly supporting both of ED and MD resonances. That is, the Mie resonances in the nanorods can be controlled by the cavity effect, and the cavity effect plays a significant role in manipulations of the spectral responses including transmittance, resonance wavelength, and bandwidth. Therefore, the cavity effect can be used to make the proposed bi-layered metasurfaces express wide colour range. For the colour generation devices, it is important to consider not only high transmittance but also wide colour gamut which is determined by spectral bandwidth and wavelength range of the expressible colours. Thus, the area of the red/green/blue (RGB) colour spaces which are formed by RGB colours expressed by the bi-layered metasurfaces (Fig. S2) is calculated as changing *w* and *l*. Figure 2f shows the calculated area of the RGB colour spaces as a function of *d*. As can be seen, the cavity effect leads to the fluctuation of the area ranging from 0.0239 to 0.0712. To obtain pure colour and wide gamut, the optimal *d* is decided as 170 nm where moderate transmittance can be obtained. As a result, the proposed structure can provide purer colour, and its RGB colour space is 144% wider than that of the nanorod layer without the nanograting (See Fig. S3).

**Figure 3:**
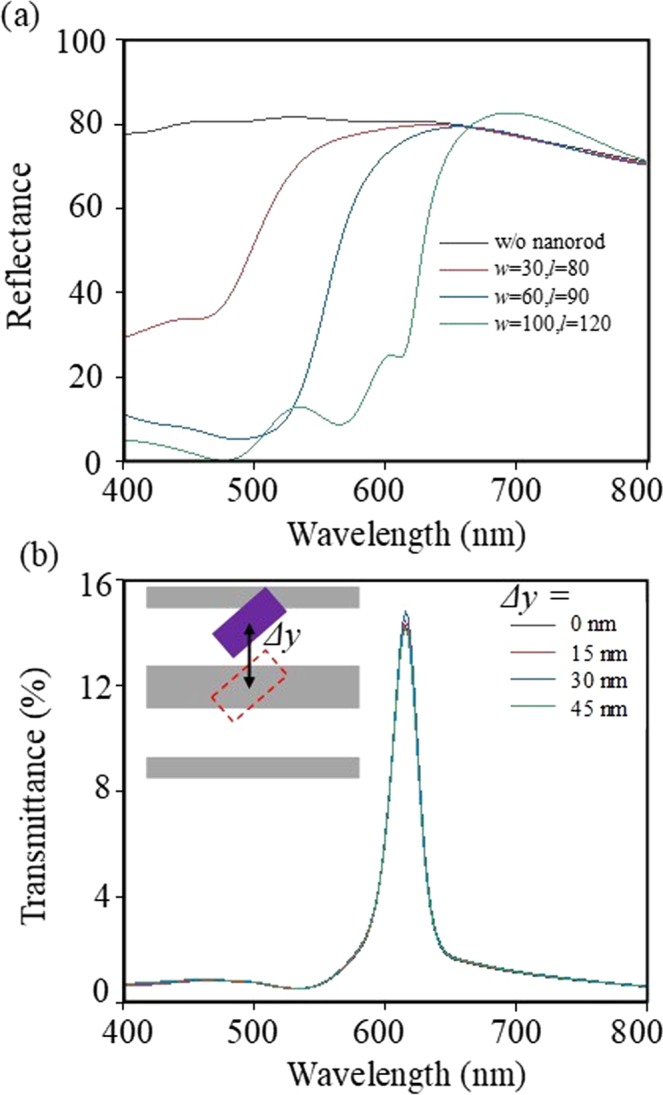
Simulation results of (**a**) reflectance spectra of the bi-layered structures with various a-Si nanorod conditions and (**b**) transmittance spectra of the bi-layered structures at the various misaligned circumstances in *y*-direction under *x*-polarized illumination. The legends in (**a**) denote the cases without nanorod (black line), nanorod with specific (*w*, *l*) values (red, blue, and green lines). The legends in (**b**) denote *y*-directional distances of misalignments.

There are two additional noteworthy points to discuss as suggested in Fig. 3. At first, when it comes to the reflection spectra of the structures, the abovementioned working principles are more clearly explained. As shown in Fig. 3a, the single Al nanograting layer reflects most of the incident *x*-polarized light over the full visible range. When the rotated nanorods is placed on the structures, however, the reflected spectrum changes with abrupt reflection decrease. It is owing to the Mie-resonant light funneling and the polarization rotation toward *y*-direction in the nanorods. Therefore, transmission through the nanograting layer increases. Secondly, since misalignment between the nanorod and grating polarizer can be an error factor in fabrication, we investigated this effect numerically (Fig. 3b). In the case of the proposed bi-layered metasurfaces, the unit cell shows translational symmetry in *x*-direction and *y*-direction with period 75 nm. Therefore, the transmittance of the proposed colour pixels (*w* = 100 nm, *l* = 120 nm) is calculated as changing the location of a-Si nanorod in *y*-direction (Δ*y*). As shown in Fig. 3b, the proposed structure is robust to the fabrication errors from the misalignment.

## Results and Discussion

**Figure 4:**
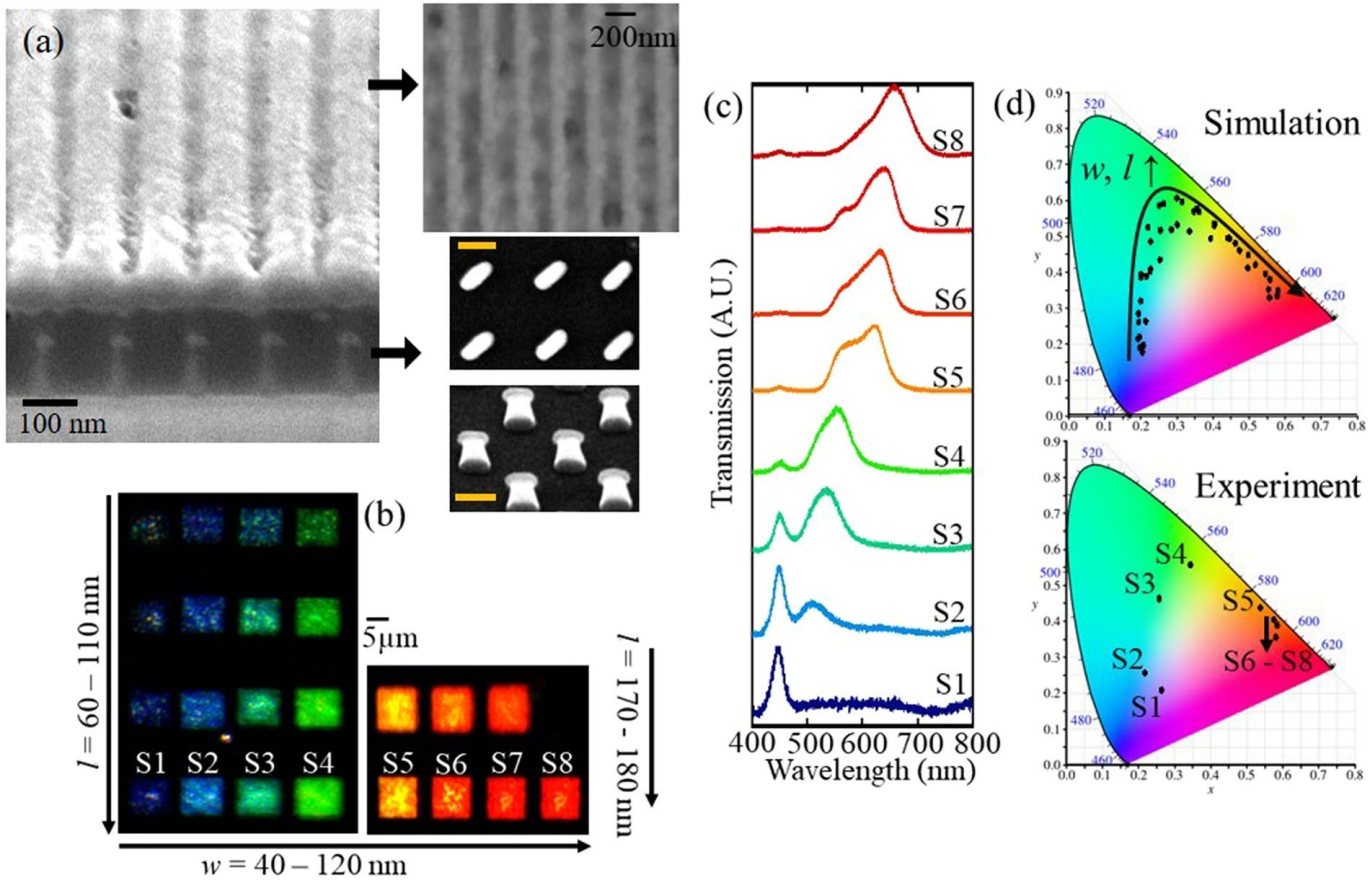
(**a**) The cross-sectional (left) and the top/oblique view (right) SEM images of the fabricated sample. The two right images under the top view of Al grating exhibit the top (upper) and oblique (lower) view SEM images of the single layer HSQ-capped silicon nanorods before stacking PMMA spacer and Al grating. The orange scale bars in the two figures denote 50 nm. (**b**) Captured images of the transmitted light from the fabricated bi-layered hybrid metasurfaces with *θ* = 45° and various *w* and *l*. (**c**) Transmittance spectra of the fabricated samples (S1–S8). (**d**) CIE chromaticity diagrams of the simulation (top) and experimental results (bottom). The simulation results are calculated for the proposed structures with various size (*w* = 30 ~ 110 nm and *l* = *w* + 10 ~ 120 nm) at *θ* = 45° and *d* = 170 nm.

The bi-layered hybrid metasurfaces are fabricated by using conventional nano patterning techniques such as electron-beam lithography and focused ion beam milling (See Methods for details of fabrication process). Here, the nanograting layer is fabricated to have a period of 200 nm, thickness of 100 nm, and width of 100 nm in order to operate as a linear polarizer over the operating wavelengths of the input source (cf. Fig. S4). Figure 4a shows SEM images of the fabricated bi-layered hybrid metasurfaces. The fabricated samples have various sizes of nanorods and *θ* = 45°, and their transmitted lights for normal incidence of *x*-polarized white LED light are captured by a camera as shown in Fig. 4b. As can be seen, the response colours of the fabricated structures exhibit a red shift as the sizes increase and express colours from blue to red. Also, it can be seen that the intensity of the transmitted light increases as *l* increases. This tendency is because the transmitted intensity of the proposed structure is proportional to the anisotropy of the nanorods as demonstrated in Eqs 3 and 4. Figure 4c shows the transmittance spectra of the fabricated samples with various size of the nanorods. The resonance wavelengths of the samples are gradually shifted from 450 nm to 660 nm when the size increases. The transmittance dips occurring around 480 nm originate from the transmittance dip of the input source (cf. Fig. S4). Also, it can be understood that the cavity effect successfully inhibits the spectral broadening from the Mie resonances in that the spectral responses maintain narrow bandwidth over the various visible wavelengths. To compare the experimental results and simulations, CIE1931 colour space of the spectral responses of the bi-layered hybrid metasurfaces is presented in Fig. 4d. At the short wavelength range, the response colours undergo a slightly low saturation due to imperfection of the fabricated nanograting layer which permits transmittance of the undesired *x*-polarized light. However, the pure colours can be generated at the long wavelength range, and the response colours of the fabricated samples can cover as wide area as the colour space of the simulation result.

**Figure 5:**
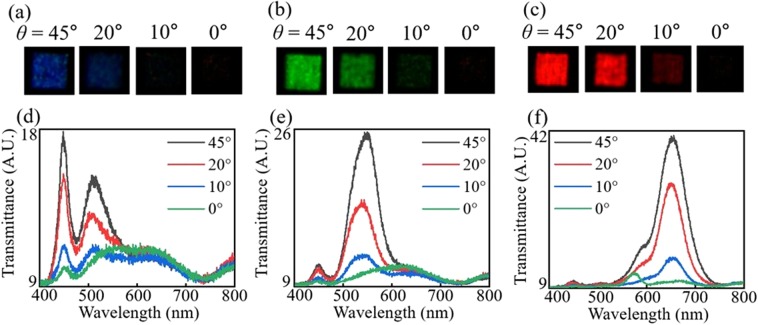
Stark intensity tuning of the bi-layered structures by rotation of the nanorods for the blue, green, and red colour pixels. (**a–c**) Captured images of the transmitted lights from the fabricated bi-layered structures with varying *θ* while keeping (**a**) *w* = 45 nm, *l* = 90, (**b**) *w* = 60, *l* = 100, (**c**) *w* = 120, *l* = 180. (**d–f**) Transmittance spectra of the fabricated samples with (**d**) *w* = 45 nm, *l* = 90, (**e**) *w* = 60, *l* = 100, (**f**) *w* = 120, *l* = 180.

Next, to independently control the intensity of the response colour, the bi-layered hybrid metasurfaces with varying *θ* while keeping the size of the nanorods are fabricated. Figure 5 shows the images and spectra of transmitted colours from the fabricated samples that generate RGB colours. As can be seen in the captured images, the intensities of the RGB samples decrease, retaining their hue and saturation, and finally the response colours become black while *θ* changes from 45° to 0°. This intensity control can be clearly shown in the spectra as shown in Fig. 5d–f. The resonance wavelengths of each RGB sample keep nearly constant regardless of *θ*, and the intensities decrease as *θ* decreases. Also, it can be observed that the spectral distortions are exacerbated at *θ* = 0° due to the imperfection of the nanograting layer leaking the unwanted *x*-polarized light. This distortion can be addressed by fabricating the homogeneous nanograting layer operating as a perfect linear polarizer. Nevertheless, it is shown that the independent control of spectral resonance and intensity can be obtained by adjusting the size and rotated angle of the dielectric nanorods, respectively.

**Figure 6:**
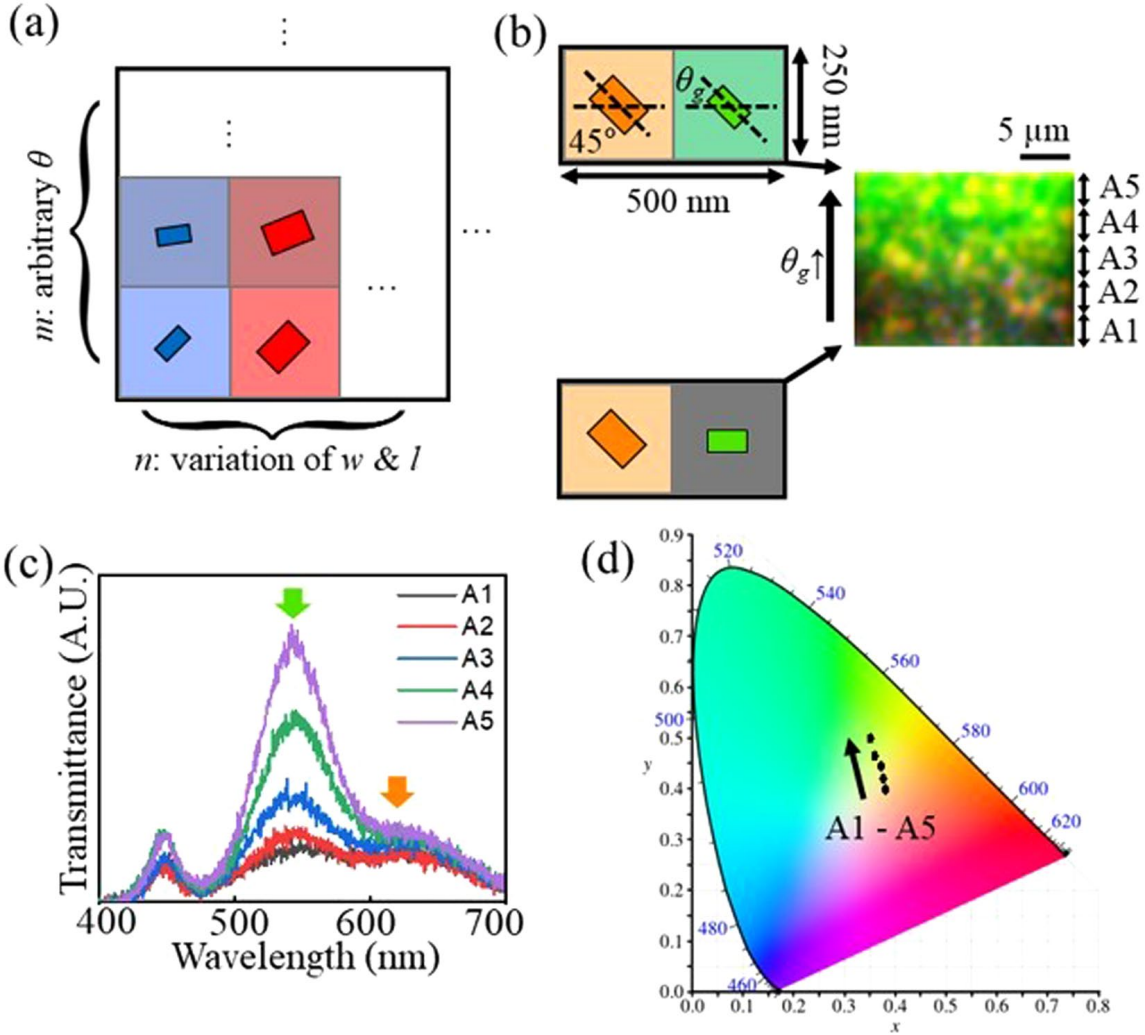
(**a**) Schematic illustration of supercell structure of the proposed meta-pixel. For simplicity, the nanograting layer is omitted in this illustration. According to colour mixing method, the response colours of the meta-pixels are decided by the mixture of the primary colours of the sub-pixels (**b**) Schematic illustration of the supercell structures of the 1 × 2 meta-pixel with orange and green sub-pixels (left), and the image of the transmitted light from the fabricated meta-pixel sample (right). The rotated angle of the orange sub-pixel is fixed at 45°, and the rotated angle of the green sub-pixel (*θ*_g_) changes from 0° to 45°. Experimentally measured (**c**) transmittance spectra and (**d**) colour space of the meta-pixel sample divided by five areas (A1–A5).

Intuitively, the aforementioned bi-layered metasurface can provide high resolution colour pixels that are achieved by placing the nanorods having various size (*w* and *l*) and *θ* at different positions. However, gradual tuning of hue, saturation, and value of the response colour is still challenging due to limited tunability of the length parameters (*w* and *l*). To overcome this low colour depth, we propose meta-pixels which can provide gradual tuning of colours based on additive colour mixing. Figure 6a demonstrates an *n* × *m* meta-pixel that is a supercell structure of the proposed bi-layered metasurfaces having various *w*, *l*, and *θ*. Each sub-pixel of the meta-pixel has different size (*w* & *l*) of the nanorods along the columns, and has arbitrary *θ* ranging from 0° to 45°. In other words, each column represents different *n* primary colours, and the total intensity of each primary colour is encoded as the sum of the *θ* in the same column. According to additive colour mixing method, the meta-pixels can express various colours inside the colour space composed of the primary colours of the sub-pixels, and the response chromaticity (hue and saturation) can be controlled by adjusting the ratio of the primary colours. Also, the value of the meta-pixel can be controlled by adjusting *θ* while keeping the ratio of the primary colours. Thus, the expressible number of colours of the meta-pixel (#*C*) can be decided by following equation:

6 $$\#C={(\frac{45^\circ }{\Delta \theta }m)}^{n}$$

where Δ*θ* indicates the variation of the rotated angle *θ* of the nanorods. The gradation of each primary colour is assigned by $\frac{45^\circ }{\Delta \theta }m$. Thus, gradation of the finally created colours becomes smoother and continuous as Δ*θ* and *m* become smaller and larger, respectively. Also, increasing the number of primary colours (*n*) is very profitable in that #C increases exponentially, and it allows to cover wide area in the colour space. As an example, the 1 × 2 meta-pixel that has orange and green as primary colours is experimentally demonstrated. The left figure in Fig. 6b shows the schematic illustration of the 1 × 2 meta-pixel. The fabricated sample (right figure in Fig. 6b) is composed of various 1 × 2 meta-pixels: The rotated angle of the green pixels *(θ*_g_) changes from 0° to 45° while that of orange keeps 45°. As shown in the captured image of the transmitted light of the fabricated meta-pixel sample, it can be observed, in spite of high magnification of the image, that the response colour changes from orange to yellow and then from yellow to green as *θ*_g_ increases. To understand this gradual colour changes more clearly, the fabricated sample is divided into five regions (A1–A5), and the transmittance spectra and CIE colour space of each region are investigated as shown in Fig. 6c,d. The resonance wavelengths of the orange and green pixels can be found at 618 nm and 542 nm, respectively. Also, it can be seen that the intensity of the resonance at 542 nm can be independently controlled by *θ*_g_ while that of the resonance at 618 nm remains nearly constant. As a result of this spectral changes, the response colours of the meta-pixels can gradually change on the straight line connecting the two primary colours (orange and green) in the colour space as shown in Fig. 6d. Compared with the method for controlling the chromaticity by adjusting the size of nanorods (cf. Fig. 6d), the proposed meta-pixels based on additive colour mixing can be used to more gradually tune the response colours, and the gradation can be improved by increasing Δ*θ* and *m*. In addition, as can be seen in Fig. 6d, the response colour of the meta-pixels can be intuitively predicted according to the additive colour mixing method. Therefore, it is a promising solution for generating high colour depth and reproducing real pictures to exploit the proposed meta-pixels.

## Conclusion

The independent control of spectral resonance wavelength and intensity with narrow bandwidth is demonstrated in the proposed bi-layered hybrid metasurfaces. Also, a potential method for generating high colour depth is presented by the proposed meta-pixels. The proposed structures have been successfully demonstrated by theoretical and experimental analysis, but some improvements are needed to apply them to practical imaging systems.

Firstly, the fabrication method for the homogeneous nanograting layer in large area is required. Actually, it is hard to fabricate the nanograting of a period of several hundred nanometers over a wide area by using FIB milling technique. Therefore, nanoimprint lithography, which has been reported to successfully fabricate high quality visible polarizers in centimeter scales, could be a promising solution for fabrication of the nanograting layer. Secondly, we discuss a potential of the bi-layered structures as dynamic colour pixels. To achieve dynamic colour pixels, it would be a possible solution to replace the a-Si nanorods with phase change materials^21–24^ that changes their refractive index according to the external stimuli. The size of the phase change material nanorods would decide the resonance wavelength, and the refractive index variation would result in the transmittance changes by manipulating the anisotropy of the rotated nanorods. Finally, the method for enhancing the efficiency of the transmittance is addressed here. The efficiency issue is originated from the absorption loss in aluminum grating and relatively low scattering intensity of the nanorods. High efficient nanograting can be accomplished by high aspect ratio and low fill factor. Moreover, in case of dielectric Mie resonator, high refractive index and low extinction coefficient is needed to enhance the scattered light from the nanorods which leads to increase in transmittance. Therefore, a-Si:H with lower extinction coefficient and similar refractive index compared to a-Si can be utilized to relieve low efficiency^25^. Also, locating multiple dielectric nanorods can be a helpful solution to increase transmittance especially at the near-blue wavelengths (shown in Fig. S5).

The bi-layered hybrid metasurfaces have significant advantages in ultrathin thickness and high colour depth generation. Therefore, we expect that the bi-layered structure can be a promising candidate for the next generation imaging systems such as flexible and foldable displays.

### Experimental section

For fabrication of the bi-layered hybrid metasurfaces, conventional nano-fabrication techniques such as focused ion beam (FIB) milling and electron beam lithography methods are used. Firstly, silicon oxide of 100 nm and a-Si thin film of 200 nm is sequentially deposited on quartz wafer substrate using PECVD (AMAT P5000, OEM group). The refractive index of the deposited a-Si film is measured by ellipsometer (J.A. Woollam, V-VASE). Next, a negative-tone resist (XR-1541-006, Dow Corning) is spin-coated, and then the nanorod patterns are transferred to the resist using electron beam lithography (JBX-6300FS, JEOL) and developed. The nanorod patterns are transferred to the a-Si layer via inductively coupled plasma etching with Cl_2_ and HBr gases. For the planarization, PMMA (950PMMA A4, Micro Chem) is spin-coated on the sample, and then 100-nm-thickness of Al is deposited using an E-gun evaporator (ZZS550-2/D, Maestech). Finally, the nanograting patterns with period of 200 nm are milled at the Al layer by FIB milling machine (Quanta 200 3D, FEI).

For the optical measurement, the fabricated bi-layered hybrid metasurfaces is illuminated from the substrate side using a broadband white light LED (MNWHL4 LED, Thorlabs). The LED source is illuminated through a broadband linear polarizer (High Contrast Glass Linear Polarizers, Edmund Optics) aligned perpendicular to the nanograting axis and 10X or 50X objective lens. The lights transmitted through the samples pass through a linear polarizer aligned parallel to the nanograting axis, and then their microscopic images are captured by camera (UCMOS05100KPA, Touptek), and the spectra are measured by spectrometer (Princeton Instruments, SpectraPro 2300).

## Supplementary information

supplementary information
