# Supplementary material for: Ultracompact meta-pixels for high colour depth generation using a bi-layered hybrid metasurface
Source: Sci Rep. 2019 Oct 25;9:15381. doi: 10.1038/s41598-019-51946-8 (PMC6814732; doi:10.1038/s41598-019-51946-8)
Supplement: Supplementary file 1 — supplementary information [file 41598_2019_51946_MOESM1_ESM.pdf]

# Ultracompact meta-pixels for high colour depth generation using a bi-layered hybrid metasurface

*Jeong-Geun Yun, Jangwoon Sung, Sun-Je Kim, Hansik Yun, Chulsoo Choi & Byoungho Lee**

Inter-University Semiconductor Research Center and School of Electrical and Computer Engineering, Seoul National University, Gwanak-Gu Gwanakro 1, Seoul 08826, Korea

*Corresponding author's E-mail: byoungho@snu.ac.kr

**S1: Derivation of transmitted light of the bi-layered hybrid metasurface**

To derive the transmitted complex amplitude of the proposed bi-layered metasurfaces $E_{\mathrm{tot}}$, Jones calculus is exploited. For normal incidence of light, the transmission and reflection matrices of the anisotropic nanorods can be expressed by $t = \begin{pmatrix} t_{xx} & 0 \\ 0 & t_{yy} \end{pmatrix}$ and $r = \begin{pmatrix} r_{xx} & 0 \\ 0 & r_{yy} \end{pmatrix}$, respectively. Those for the rotated nanorods ($t(\theta)$ and $r(\theta)$) can be easily derived using rotation matrix $R = \begin{pmatrix} \cos\theta & \sin\theta \\ -\sin\theta & \cos\theta \end{pmatrix}$ and expressed as following equations

$$t(\theta) = R^{-1}tR = \begin{pmatrix} t_{xx}\cos^2\theta + t_{yy}\sin^2\theta & \cos\theta\sin\theta(t_{xx}-t_{yy}) \\ \sin\theta\cos\theta(t_{xx}-t_{yy}) & t_{xx}\sin^2\theta + t_{yy}\cos^2\theta \end{pmatrix}, \quad \text{(S1)}$$

$$r(\theta) = R^{-1}rR = \begin{pmatrix} r_{xx}\cos^2\theta + r_{yy}\sin^2\theta & \cos\theta\sin\theta(r_{xx}-r_{yy}) \\ \sin\theta\cos\theta(r_{xx}-r_{yy}) & r_{xx}\sin^2\theta + r_{yy}\cos^2\theta \end{pmatrix}. \quad \text{(S2)}$$

As discussed on the main manuscript, the transmitted light of the proposed structure is resulted from superposition of the direct-transmission and multiple reflection components (see Fig. 1(c)). Therefore, Jones vector of the transmitted light can be derived from the following:

$$e^{ikn_1d}t_g t(\theta) + e^{i3kn_1d}t_g r(\theta) r_g t(\theta) + e^{i5kn_1d}t_g r(\theta) r_g r(\theta) r_g t(\theta) + \cdots, \quad \text{(S3)}$$

where $k$ is free space wavenumber, $n_1$ is refractive index of the spacer medium, $d$ is the distance between the nanograting and nanorod layer, and $t_g$ and $r_g$ indicate the transmission and reflection matrix of the nanograting layer. For simplicity, we assume that the nanograting layer acts like a perfect polarizer over the entire visible frequency range. This means that $t_g = \begin{pmatrix} 0 & 0 \\ 0 & 1 \end{pmatrix}$ and $r_g = \begin{pmatrix} 1 & 0 \\ 0 & 0 \end{pmatrix}$ are assumed in this derivation. Using Equations (S1-S3), the transmitted Jones vector of the proposed bi-layered metasurface can be derived as following:

$$e^{i\varphi}\begin{pmatrix} 0 & 0 \\ sc\left[t_{xx} - t_{yy} + e^{i2\varphi}(r_{xx} - r_{yy})(t_{xx}c^2 + t_{yy}s^2)\sum_{j=1}^{\infty} A^{j-1}\right] & t_{xx}s^2 + t_{yy}c^2 + e^{i2\varphi}(t_{xx} - t_{yy})(r_{xx} - r_{yy})s^2c^2\sum_{j=1}^{\infty} A^{j-1} \end{pmatrix}, \quad \text{(S4)}$$

where $\varphi$ is $kn_1d$, $A$ is $e^{i2kn_1d}(r_{xx}\cos^2\theta + r_{yy}\sin^2\theta)$, $s$ and $c$ indicate $\sin\theta$ and $\cos\theta$, respectively. Since the multiple reflection terms of the higher order ($j$ > 1) are relatively smaller, it is sufficient to understand the transmission tendency only with the non-reflection and the first order ($j$ = 1) of the multiple reflection term. Thus, for $x$-polarized incident light, $E_{tot}$ can be derived as following equation:

$$E_{tot} = e^{ikn_1d}\sin\theta\cos\theta\left[t_{xx} - t_{yy} + e^{i2kn_1d}(r_{xx} - r_{yy})(t_{xx}\cos^2\theta + t_{yy}\sin^2\theta)\right]\bar{y}\,. \quad \text{(S5)}$$

**S2: The calculation of the area of the RGB colour spaces**

For the wide gamut colour generation, not only the spectral bandwidth but also the resonance wavelength should be considered. To optimize the $d$, we calculate the colour space of the bi-layered structures having various $w$ and $l$. Figure S2 shows the CIE 1931 colour space of the bi-layered structures with $d$ = 170 nm. Then, among the various nanorod sizes, the three specific structures generating RGB colours with the highest purity (highly saturated) are selected to calculate the area. Here, we define the area of the RGB colour space as the area of the triangle formed by these RGB structures. According to additive colour mixing method, every points inside this RGB colour space can be expressed by the combinations of the RGB colours, and thus the wider RGB colour space becomes, the more the various colour can be expressed. Therefore, comparing the RGB colour spaces having various $d$ could be exploited to evaluate the effect of the cavity in the colour gamut of the proposed bi-layered metasurfaces.

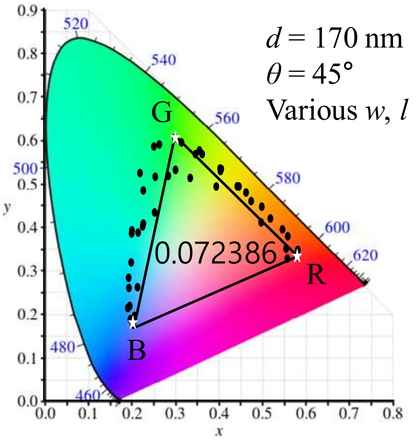


**Figure S2.** Calculated CIE chromaticity diagrams of the bi-layered hybrid metasurfaces as changing $w$ and $l$. The distance $d$ and the rotated angle of the nanorods $\theta$ are set to 170 nm and 45°, respectively. The bi-layered structures with the highest purity RGB colours are indicated by white solid stars. Each RGB structure has various size of $w = 30$ nm, $l = 50$ nm (blue), $w = 60$ nm, $l = 90$ nm (green), $w = 110$ nm, $l = 120$ nm (red).

**S3: The bi-layered hybrid metasurface versus nanorod layer without the nanograting**

To confirm the wide gamut of the bi-layered hybrid metasurfaces, we calculate the area of the RGB colour space for our structure and the nanorod layer without the grating layer as shown in Fig. S3. It is noticeable that the response colour of the bi-layered structure provides pure colours especially at low wavelength range. In addition, the bi-layered structure can express more various colours at shorter (around 480 nm) and longer (around 605 nm) wavelengths, and thus the area of RGB primary colour space of the bi-layered structures can cover wider area (0.072386) than that of the nanorod layer without the grating (0.050293).

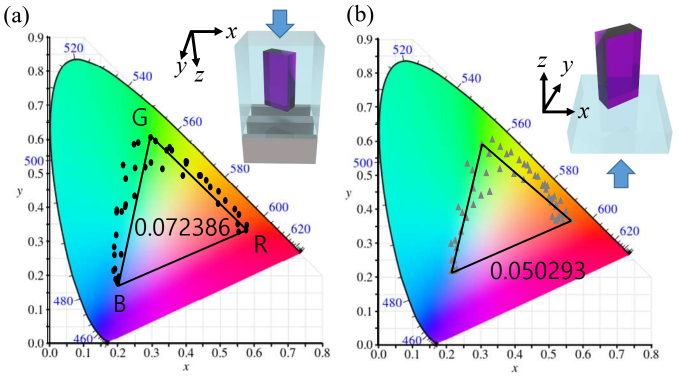


**Figure S3.** Calculated CIE chromaticity diagrams of (a) the bi-layered hybrid metasurfaces and (b) the nanorod layers without the nanograting as changing $w$ and $l$.

**S4. The spectrum of the input white LED**

For experimental demonstration of the proposed bi-layered hybrid metasurfaces, the white LED light is used as an input source (MNWHL4 LED, Thorlabs). As shown in Fig. S4, the spectrum of the LED source exhibits broad wavelengths. Also, it is noticeable that the transmittance deep is observed at 480 nm due to the narrow bandwidth of the blue light composing the white LED.

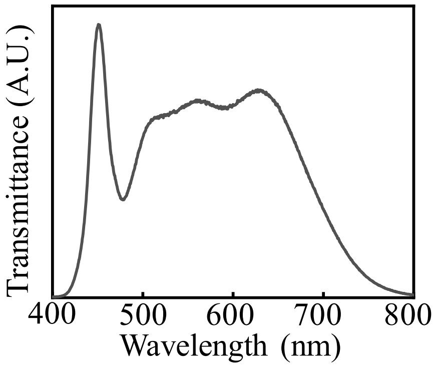


**Figure S4.** Transmittance spectrum of the white LED light source.

**S5. Low transmittance issue**

In order to enhace the scattering intensity of the nanarods, replacing the material with high refractive index and low extinction coefficient one such as a-Si:H and expoiting double nanorods structure can be considered. As shown in Fig. S5, the transmittance of the double rods structure with a-Si:H is dramatically enhanced.

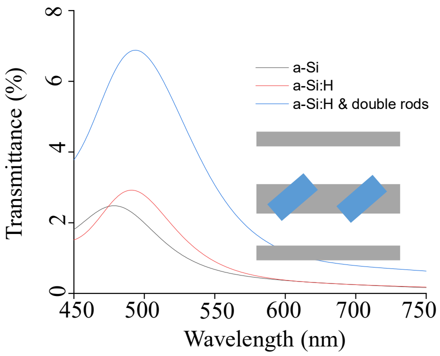


**Figure S5.** Transmittance spectrum of the various nanorods ($w$ = 30 nm, $l$ = 80 nm, $d$ = 50 nm). The inset indicates the schematic illustration of the double rod structure.
